# Urinary tetrahydroaldosterone is associated with circulating FGF23 in kidney stone formers

**DOI:** 10.1007/s00240-022-01317-2

**Published:** 2022-02-24

**Authors:** Matthias B. Moor, Nasser A. Dhayat, Simeon Schietzel, Michael Grössl, Bruno Vogt, Daniel G. Fuster

**Affiliations:** 1 Department of Nephrology and Hypertension, Inselspital, Bern University Hospital, Freiburgstrasse 15, 3010 Bern, Switzerland; 2 Department of Biomedical Research, University of Bern, Bern, Switzerland

**Keywords:** FGF23, Renin–angiotensin system, Aldosterone, Tetrahydroaldosterone, Steroid metabolite

## Abstract

The spectrum of diseases with overactive renin–angiotensin–aldosterone system (RAS) or elevated circulating FGF23 overlaps, but the relationship between aldosterone and FGF23 remains unclarified. Here, we report that systemic RAS activation sensitively assessed by urinary tetrahydroaldosterone excretion is associated with circulating C-terminal FGF23. We performed a retrospective analysis in the Bern Kidney Stone Registry, a single-center observational cohort of kidney stone formers. Urinary excretion of the main aldosterone metabolite tetrahydroaldosterone was measured by gas chromatography–mass spectrometry. Plasma FGF23 concentrations were measured using a C-terminal assay. Regression models were calculated to assess the association of plasma FGF23 with 24 h urinary tetrahydroaldosterone excretion. We included 625 participants in the analysis. Mean age was 47 ± 14 years and 71% were male. Mean estimated GFR was 94 ml/min per 1.73 m^2^. In unadjusted analyses, we found a positive association between plasma FGF23 and 24 h urinary tetrahydroaldosterone excretion (*β*: 0.0027; *p* = 4.2 × 10^–7^). In multivariable regression models adjusting for age, sex, body mass index and GFR, this association remained robust (*β*: 0.0022; *p* = 2.1 × 10^–5^). Mineralotropic hormones, 24 h urinary sodium and potassium excretion as surrogates for sodium and potassium intake or antihypertensive drugs did not affect this association. Our data reveal a robust association of RAS activity with circulating FGF23 levels in kidney stone formers. These findings are in line with previous studies in rodents and suggest a physiological link between RAS system activation and FGF23 secretion.

**Supplementary Information:**

The online version contains supplementary material available at 10.1007/s00240-022-01317-2.

## Introduction

Cardiovascular complications are a major cause of the high morbidity and mortality in chronic kidney disease. Both cardiovascular and kidney disease have a broad spectrum of etiologies and clinical presentation, but during progression of each disease, two hallmarks are an activation of the renin–angiotensin–aldosterone system (RAS), and a rise in serum concentrations of fibroblast growth factor 23 (FGF23) [1].

In patients with advanced heart failure, a direct association between plasma aldosterone and FGF23 was observed, but almost half of the patients were on aldosterone antagonists [2]. In larger and more recent studies, however, this observation could not be reproduced [3] [4]. Only indirect connections between RAS and FGF23 are available in these studies: hemodynamic intolerance of titrating RAS inhibitors up to target dosage was associated with higher FGF23 concentrations [4]. In hemodialysis patients, ultrafiltration volume as a surrogate for volume status positively associates with FGF23 concentrations [5].

We and others have previously shown in rodent models that salt depletion not only caused RAS activation but also increased circulating FGF23 [6, 7]. Vice versa, treatment of rodents with FGF23 causes volume expansion and decreased serum and urinary aldosterone [8]. Pharmacological inhibition of excessive FGF23 in transgenic mice reverses the suppression of aldosterone [9]. These experimental results strongly support a direct link between RAS and FGF23.

We hypothesized that the difficulty to reproduce the reported association between aldosterone and FGF23 concentration in humans could arise from heterogeneity of the analyzed patient populations, a variability of the assays used [10], from the circadian rhythmicity of aldosterone secretion [11, 12], or from a combination of the above.

Quantification of steroid hormones in timed urine collections allows sensitive determination of secreted hormone quantities over time [13]. Based on a well-characterized single-center registry of patients with kidney stones, we report that RAS activity surveyed by the urinary excretion rate of metabolite tetrahydroaldosterone [14] is independently associated with circulating FGF23.

## Materials and methods

### Study population

The Bern Kidney Stone Registry (BKSR) is a single-center, observational cohort of kidney stone formers at the Department of Nephrology and Hypertension, Bern University Hospital, Bern, Switzerland. The BKSR adheres to the Declaration of Helsinki and was approved by the ethical committee of the Kanton of Bern (approval # BE 95/06). Inclusion criteria for the BKSR are (i) written informed consent, (ii) age ≥ 18 years, and (iii) at least one past stone episode. All variables employed for study analyses were verified by manual review of the original patient charts. In this study, we included BKSR participants who had urinary steroid profiles available and who had 24 h urine creatinine excretion that was within the 2.5th and 97.5th percentile of expected creatinine excretion [15]. Among the 1422 BKSR participants, 681 had a urinary steroid profile analysis available. Among these, 56 BKSR participants had 24 h urine creatinine excretion below the 2.5th or above the 97.5th percentile [15] and were excluded. Of the remaining 625 BKSR participants, 14 had no available urinary tetrahydroaldosterone analysis and 297 no measurement of FGF23. A final sample of 314 participants was included in the current study.

### Data collection and measurements

Patients were instructed to collect two 24 h urine collections on a random outpatient diet and a spot urine and a fasting blood sample were obtained in the morning after the second of the 24 h urine collections. Plasma C-terminal FGF23 was measured at the laboratory of TECOmedical AG (Sissach, Switzerland) by the second-generation C-terminal assay (Immutopics, San Clemente, CA, USA) with plasma initially frozen after sampling and stored at − 80 °C. C-terminal FGF23 was chosen due to its lower diurnal variability in comparison to intact FGF23 [16]. All other urine and blood parameters were analyzed at the Central Chemistry Laboratory of the Bern University Hospital directly after sampling, as described [17]. Assay characteristics for the measurements of FGF23, PTH, 25(OH)D and 1,25(OH)_2_D were previously described [18]. The glomerular filtration rate (GFR) was estimated using the creatinine-based Chronic Kidney Disease Epidemiology Collaboration (CKD-EPI) 2009 equation [19]. Urinary creatinine excretion was used as the criterion for completeness of 24 h urine collections in reference to a population with normal renal function [20, 21]. Percentiles 2.5 and 97.5 of 24 h creatinine excretion were calculated for each 24 h urine collection using linear regression [22]. Completeness of 24 h urine collections was assumed if the total 24 h creatinine excretion was within percentiles 2.5 and 97.5, and the 56 samples not within creatinine percentiles 2.5 and 97.5 were excluded. The mean value of both 24 h urine collections was used for the calculation of 24 h urinary excretion. Tubular maximum reabsorption of phosphate to glomerular filtration rate (TmP/GFR) was calculated as previously described [23]. Diabetes was defined as presence of reported or treated diabetes and/or fasting glycemia ≥ 7 mmol/L (≥ 126.13 mg/dL). Hypertension was defined by presence of any of: anti-hypertensive therapy prescribed, a mean systolic blood pressure ≥ 140 mmHg or a mean diastolic blood pressure ≥ 90 mmHg.

### Quantification of urinary tetrahydroaldosterone by GC–MS

Urinary excretion of urinary tetrahydroaldosterone in μg/24 h was assessed in-house using an established GC–MS method as previously described [13, 24, 25]. Urine sample preparation consisted of pre-extraction on a Sep-Pak C18 column with a recovery standard medroxyprogesterone, followed by enzymatic hydrolysis using sulfatase and *β*-glucuronidase/arylsulfatase and extraction on Sep-Pak C18 cartridge. Steroids were derivatized using methoxyamine HCl 2% in pyridine at 60 °C for 1 h after adding Stigmasterol and 3*β*5*β*-TH-aldosterone standards, followed by derivatization with Tri-methyl-silylimidazole (TMSI) at 100 °C for 16 h, and gel filtration on a Lipidex 5000 column. Samples were quantified using mass spectrometric analysis on a gas chromatograph 7890A coupled to a mass-selective Hewlett-Packard 5975C detector (both from Agilent Technologies, La Jolla, California, USA). Quality control of the method was ensured by comparison with parallel measurement of samples from healthy volunteers, and by monthly participation in external quality control as previously reported [25].

### Statistical analysis

All statistical analyses were conducted using R software, version 4.0.4 (R Project for Statistical Computing, Vienna, Austria) [26]. The distribution of continuous variables was visually inspected. For C-terminal FGF23, in a univariable linear model with urinary tetrahydroaldosterone excretion, residuals were not normal distributed in assessment using the olsrr package (SFig 1a–b). Therefore, C-terminal FGF23 underwent log transformation to improve distribution toward normality of residuals in statistical models. Statistical two-sample comparison was obtained using a two-sided Welch two-sample *t* test, and *p* values < 0.05 were considered statistically significant.

To assess the associations of log C-terminal FGF23 with urinary tetrahydroaldosterone, we computed a univariable linear model. This association of log C-terminal FGF23 with urinary tetrahydroaldosterone was further analyzed by eight multivariable linear models adjusting for sex, age, body mass index (BMI), eGFR (multivariable model 1), model 1 parameters and parathyroid hormone, 25OH-vitamin D and 1.25(OH)2-vitamin D (multivariable model 2); model 1 parameters and 24 h sodium and potassium excretion (multivariable model 3). Multivariable linear model 4 was adjusted for model 1 parameters and seven classes of concurrently prescribed antihypertensive drugs (loop diuretics, thiazide diuretics, potassium-sparing diuretics, RAS inhibitors, alpha1 blockers, beta blockers, and calcium antagonists. Four further multivariable models 1b to 4b were similarly calculated using the same set of parameters as before except including mGFR instead of eGFR.

Selected regression models were visualized using the visreg package to show entire models. Further, visreg was used to display effects of categorical subgroups and continuous predictor variables on the relationship between log C-terminal FGF23 and urinary tetrahydroaldosterone, while maintaining effects of all other co-variables in the model constant [27].

## Results

A total of 625 Bern Kidney Stone Registry (BKSR) participants met the predefined eligibility criteria and were included in this study (for details see Materials and Methods section). Clinical characteristics of study participants are shown in Table 1. Age of participants varied from 18 to 76 years, mean age ± SD was 47 ± 14 years and 71% were men. Arterial hypertension was present in 38% and diabetes mellitus in 9% of participants, respectively. The mean estimated GFR (eGFR; creatinine-based CKD-EPI equation) was 94 ± 21 ml/min per 1.73 m^2^. Only 6% of the study population had an eGFR < 60 ml/min per 1.73 m^2^ and thus met the definition of CKD.

**Table 1:** Population characteristics

Parameter	*n*	%, mean ± SD or median (25th–75th percentile)
Age	314	46.8 ± 14.1
Sex (male)	228	72.6
Height (cm)	314	173.4 (165.4–179.6)
Weight (kg)	314	80.9 ± 16.9
BMI (kg/m^2^)	314	27.1 ± 4.9
Blood analyses		
Creatinine (µmoL/L)	314	79.7 ± 21.0
eGFR CKD-EPI (mL/min/1.73 m^2^ BSA)	314	95.5 ± 21.2
Measured GFR (mL/min/1.73 m^2^ BSA)	314	111.3 ± 27.3
Hypertension	113	36
Diabetes melitus	32	10.2
25OH D3 (nmol/L)	296	38.0 (24.0–51.0)
1,25OH (pmol/L)	312	83.5 (63.0–110.3)
cFGF23 (RU/mL)	314	70.4 (54.2–96.4)
Alkaline phosphatase (U/L)	312	64.0 (54.0–74.0)
PTH (pg/mL)	311	40.0 (31.0–50.0)
Albumin (g/L)	309	40.0 ± 3.2
Calcium, albumin-corrected (mmol/L)	309	2.35 ± 0.11
Ionized calcium, ph-corrected (mmol/L)	305	1.21 ± 0.04
Phosphate (mmol/L)	313	0.98 ± 0.17
Magnesium (mmol/L)	311	0.83 ± 0.07
Sodium (mmol/L)	311	140.6 ± 2.1
Potassium (mmol/L)	313	3.84 ± 0.28
Urinary analyses		
24 h urinary volume (mL)	314	2006 (1438–2595)
Creatinine (µmol/24 h)	314	13,975 ± 4359
Tetrahydroaldosterone (µg/24 h)	314	20.1 (14.3–31.1)
Medication		
Loop diuretics	6	1.9
Thiazide diuretics	33	10.5
K-sparing diuretics	1	0.3
Beta blockers	26	8.3
RAS inhibitors	54	17.3
Calcium antagonists	11	3.5
Alpha1-blockers	8	2.6

*SD* standard deviation, *BSA* body surface area, *RAS* renin angiotensin system

In BKSR participants with biobank plasma available, plasma FGF23 concentration was measured by ELISA, as previously described (*N* = 322) [23]. There were no significant differences between participants with and without FGF23 measurements when considering age (*p* = 0.21), eGFR (*p* = 0.82) or urinary tetrahydroaldosterone (*p* = 0.13). The distribution of plasma FGF23 was normal with long right tail in both men and women with a median level of 68.7 and 91.0 relative units/ml (interquartile range 36.0 and 66.9) in men and women, respectively (Supplemental Fig. 1A andB). We found a non-significant trend to higher plasma FGF23 concentrations in women (Supplemental Fig. 1B), but our dataset consisted largely of men—representing the sex difference in incidence of kidney stones [28].

**Fig. 1:**
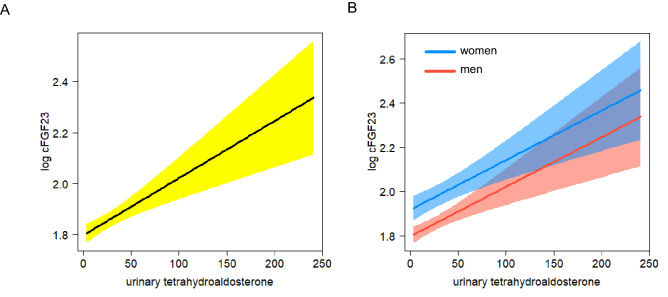
Association of urinary tetrahydroaldosterone with plasma FGF23 in kidney stone formers. **A** and **B** display visualizations of a multivariable linear model in which urinary tetrahydroaldosterone (µg/24 h) associated with log-transformed FGF23 [log FGF23 RU/mL]. The model was adjusted for age, sex, eGFR and BMI. **B** depicts individual regression lines for each sex separately. Color-shaded areas indicate 95% confidence intervals

We aimed to determine whether the daily urinary excretion of the main aldosterone metabolite tetrahydroaldosterone associates with log-transformed C-terminal FGF23 plasma concentrations. Because of non-normal distributions of model residuals in preliminary analyses, all statistical modeling was performed using log-transformed C-terminal FGF23 values as dependent variable. A strong positive correlation between tetrahydroaldosterone and log-transformed C-terminal FGF23 (*β*: 0.0027356; 95% CI 0.0016945–0.00377668; *p* = 4.19 × 10^–7^) was observed in univariable analysis (Table 2).

**Table 2:** Univariable and multivariable models for FGF23

Covariable for log cFGF23	*n*	*β*	Lower 95% CI	Upper 95% CI	*p* value
	Univariable model				
Urinary tetrahydroaldosterone per log cFGF23 RU/mL increase	314	0.0027356	0.0016945	0.00377668	**4.19E-07**
	Multivariable model 1				
Urinary tetrahydroaldosterone per log cFGF23 RU/mL increase	314	0.0022452	0.06272641	0.17804995	**0.0000213**
	Multivariable model 2				
Urinary tetrahydroaldosterone per log cFGF23 RU/mL increase	295	0.0024177	0.00133124	0.00350408	**1.67E-05**
	Multivariable model 3				
Urinary tetrahydroaldosterone per log cFGF23 RU/mL increase	314	0.0022255	0.00118121	0.00326976	**3.60E-05**
	Multivariable model 4				
Urinary tetrahydroaldosterone per log cFGF23 RU/mL increase	313	0.0021891	0.00114406	0.00323414	**4.87E-05**

Multivariable model 1 adjusted for age, sex, body mass index and eGFR. Model 2 adjusted for age, sex, eGFR, body mass index, parathyroid hormone, 25OH-vitamin D and 1.25(OH)_2_-vitamin D. Model 3 adjusted for age, sex, body mass index, eGFR, and 24 h urine sodium and potassium excretion. Model 4 adjusted for parameters of model 3 and anti-hypertensives (alpha1 blockers, beta blockers, calcium antagonists, potassium-sparing diuretics, loop diuretics, thiazides and renin angiotensin system inhibitors)

Significance was assumed at *p* < 0.05 without adjustment for multiplicity (in bold)

Several confounding factors can affect plasma FGF23. We therefore adjusted the association between tetrahydroaldosterone excretion and plasma FGF23 for sex, eGFR, age and body mass index (BMI) using a multivariable linear regression model. In this model, the association of tetrahydroaldosterone with plasma FGF23 remained robust (multivariable model 1, Table 2). Figure 1A shows a visualization of this model’s estimates. There were no diverging trends across different sexes reflected by the absence of a significant interaction term between urinary tetrahydroaldosterone and sex in multivariable analysis (Fig. 1B). No significant modification of this association was observed by age (Supplemental Fig. 2A), BMI (Supplemental Fig. 2B) and eGFR (Supplemental Fig. 2C).

Because parathyroid hormone (PTH) and vitamin D are important modulators of FGF23 secretion [29, 30], we performed a multivariable linear regression analysis with adjustment for PTH, 1,25(OH)2-vitamin D and 25OH-vitamin D. These adjustments did not affect the association between urinary tetrahydroaldosterone and circulating FGF23 (Table 2, Supplemental Fig. 2D–F). Moreover, to investigate a physiological consequence of FGF23 actions, we determined if urinary tetrahydroaldosterone is associated with TmP/GFR. In an exploratory analysis adjusted for age, sex, BMI and eGFR, there was a negative association between TmP/GFR and urinary tetrahydroaldosterone with *β* − 17.3 (95% CI − 33.2; − 1.4), *p* = 0.03. This finding remained robust (*β* − 19.3; 95% CI − 34.7; − 3.8 and *p* = 0.01) when PTH was additionally added in the model.

**Fig. 2:**
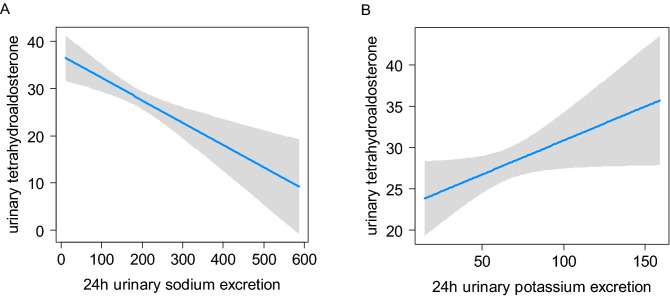
Association of 24 h urinary sodium and potassium with 24 h urinary tetrahydroaldosterone excretion. Univariable association between urinary (**A**) sodium and **B** potassium excretion (mmol/24 h) and urinary excretion of tetrahydroaldosterone (µg/24 h). Gray shaded areas represent 95% confidence intervals

Dietary intake of sodium or potassium is both associated with a decrease in circulating FGF23 independent from PTH or 25-hydroxy vitamin D3 [31]. We therefore investigated if 24 h urinary sodium and potassium excretion, well-established markers of sodium and potassium intake [32, 33], influence the association between tetrahydroaldosterone excretion and plasma FGF23. First, we determined the association between 24 h urinary sodium and potassium excretion and 24 h urinary tetrahydroaldosterone excretion. In univariable regression analysis, 24 h urinary sodium excretion showed an inverse association with urinary tetrahydroaldosterone excretion (*β* − 0.047 and *p* = 1.9 × 10^–4^) as expected [34] (Fig. 2A). In contrast, 24 h urinary potassium excretion correlated positively with urinary tetrahydroaldosterone excretion with *β*: 0.082 and *p* = 0.047, as expected [35] (Fig. 2B). However, both 24 h urinary sodium and potassium excretion did not significantly affect the association between urinary tetrahydroaldosterone excretion and plasma FGF23 adjusted for age, sex, BMI and eGFR (multivariable model 3, Table 2 and Supplemental Figs. 3A–B).

Next, we estimated that activation of the RAS could underlie a bias from concurrent medication of diuretics or RAS inhibitors that would affect volume state. We introduced 7 classes of concurrently prescribed antihypertensive medications as confounding variables in a third multivariable linear model. The association between urinary tetrahydroaldosterone and plasma FGF23 remained unaffected after inclusion of loop diuretics (Supplemental Fig. 4A), thiazide diuretics (Supplemental Fig. 4B), potassium-sparing diuretics (Supplemental Fig. 4C), RAS inhibitors (Supplemental Fig. 4D), alpha-blockers (Supplemental Fig. 4E), beta blockers (Supplemental Fig. 4F), and calcium antagonists (Supplemental Fig. 4G) in multivariable linear model 4 (Table 2).

Finally, we assessed the degree of the outlined correlation using measured GFR (mGFR) by creatinine clearance instead of eGFR (CKD-EPI) for kidney function adjustment. Because of the exclusion of participants with urine collections outside the 2.5th and 97.5th percentile of expected creatinine excretion [15], the association between mGFR and eGFR (CKD-EPI) in our population was comparable to associations between mGFR and eGFR reported in previous studies [36, 37] with *β*: 1.02105, *p* = 2 × 10^–16^ and R^2^ of 0.59 (Supplemental Fig. 5). Adjustment for mGFR instead of eGFR did not alter any of the observed associations with plasma FGF23 in multivariable models (Supplemental Table).

## Discussion

In the present study, we found that the major urinary metabolite of aldosterone, tetrahydroaldosterone, quantified in 24 h urines by a sensitive and specific GC–MS assay [24] is positively associated with FGF23 concentrations in plasma of kidney stone formers. This association was robust with regard to potential confounders. Our analyses provide a long-sought building block to strengthen a pathophysiological concept that integrates both RAS activation and excess in FGF23 as a common final stretch in heart and kidney disease [1].

This observation has previously been provided in a first study by Imazu et al. [2] that is potentially biased by a wide use of aldosterone antagonists. It has furthermore been supported by a myocardial tissue analysis in human autopsies [38] and experimental data in rodents [6–9]. All further available data in humans are at best indirect hints [3–5] or use very limited patient numbers [39].

We confirm the earlier study by Imazu et al. in patients with heart failure patients and/or early kidney disease of whom 43% were prescribed aldosterone antagonists [2]. In that study, plasma aldosterone positively associated with intact FGF23 concentrations irrespective of eGFR values [2]. In the present work, however, we investigated a different patient population, we used a different and much more sensitive aldosterone surrogate, and we employed a C-terminal FGF23 assay that is more robust for predicting adverse outcomes than intact FGF23 [40]. We also show the functional importance of our findings by displaying the negative association between urinary tetrahydroaldosterone and renal phosphate excretion using TmP/GFR. Another strength of the current study is the thorough assessment of the potential confounding effect of antihypertensive drug classes on the association between RAS activation and FGF23. We found no evidence that the observed effects are due to prescribed antihypertensive medications.

Further, our analyses demonstrate the suitability of the urinary metabolite tetrahydroaldosterone to assess clinical context or biomarkers associated with RAS activation. This has been previously suggested by others and even proposed as a screening tool for primary hyperaldosteronism [41]. For other steroid hormones also excreted in the urine, we have previously analyzed the current patient collective of kidney stone formers and have found associations of sex hormones with lithogenic factors, such as calcium, citrate and oxalate excretion [17].

The present study has some limitations. With regard to the population studied, the present results are limited to stone formers. Stone formers constitute a somewhat heterogeneous population often exhibiting disadvantageous life styles and chronic diseases predisposing for stone formation with varying levels of kidney function.

Whether the association between aldosterone activation and plasma FGF23 exists beyond kidney stone formers remains to be validated, e.g., in populations of healthy volunteers and of patients with chronic kidney disease or congestive heart failure.

Further, some variables, such as plasma renin activity or plasma aldosterone, were not available in the current study. However, results from a comparative study showed that urinary tetrahydroaldosterone excretion is the most reliable parameter to predict primary hyperaldosteronism [41]. Another limitation is the relatively small proportion of patients on diuretics, precluding us to safely estimate their potential effects on C-terminal FGF23 beyond the current dataset. Finally, by the nature of the cross-sectional study design, we were unable to assess clinical outcomes associated with excessive FGF23 and RAS activation.

In conclusion, our data reveal a robust association of RAS activity with circulating FGF23 in kidney stone formers. These findings are in line with previous studies in rodents and suggest a physiological link between the RAS system activation and FGF23 secretion.

## Supplementary Information

Below is the link to the electronic supplementary material.

Supplementary file1 (PDF 363 KB)

## Data Availability

Data are available from the authors on request.

## References

[1] de Seigneux S, Martin P-Y (2016). Phosphate and FGF23 in the renoprotective benefit of RAAS inhibition. Pharmacol Res.

[2] Imazu M, Takahama H, Asanuma H, Funada A, Sugano Y, Ohara T, Hasegawa T, Asakura M, Kanzaki H, Anzai T, Kitakaze M (2014). Pathophysiological impact of serum fibroblast growth factor 23 in patients with nonischemic cardiac disease and early chronic kidney disease. Am J Physiol Heart Circ Physiol.

[3] Akhabue E, Vu T-HT, Vaidya A, Michos ED, de Boer IH, Kestenbaum B, Allison M, Szklo M, Ouyang P, Yancy CW, Wolf M, Isakova T, Carnethon MR (2019). Fibroblast growth factor-23, heart failure risk, and renin-angiotensin-aldosterone-system blockade in hypertension: the MESA study. Am J Hypertens.

[4] Ter Maaten JM, Voors AA, Damman K, van der Meer P, Anker SD, Cleland JG, Dickstein K, Filippatos G, van der Harst P, Hillege HL, Lang CC, Metra M, Navis G, Ng L, Ouwerkerk W, Ponikowski P, Samani NJ, van Veldhuisen DJ, Zannad F, Zwinderman AH, de Borst MH (2018). Fibroblast growth factor 23 is related to profiles indicating volume overload, poor therapy optimization and prognosis in patients with new-onset and worsening heart failure. Int J Cardiol.

[5] Humalda JK, Riphagen IJ, Assa S, Hummel YM, Westerhuis R, Vervloet MG, Voors AA, Navis G, Franssen CFM, de Borst MH, NIGRAM Consortium (2016). Fibroblast growth factor 23 correlates with volume status in haemodialysis patients and is not reduced by haemodialysis. Nephrol Dial Transplant Off Publ Eur Dial Transpl Assoc-Eur Ren Assoc.

[6] Pathare G, Anderegg M, Albano G, Lang F, Fuster DG (2018). Elevated FGF23 levels in mice lacking the thiazide-sensitive NaCl cotransporter (NCC). Sci Rep.

[7] Zhang B, Umbach AT, Chen H, Yan J, Fakhri H, Fajol A, Salker MS, Spichtig D, Daryadel A, Wagner CA, Föller M, Lang F (2016). Up-regulation of FGF23 release by aldosterone. Biochem Biophys Res Commun.

[8] Andrukhova O, Slavic S, Smorodchenko A, Zeitz U, Shalhoub V, Lanske B, Pohl EE, Erben RG (2014). FGF23 regulates renal sodium handling and blood pressure. EMBO Mol Med.

[9] Xiao Z, Liu J, Liu S-H, Petridis L, Cai C, Cao L, Wang G, Chin AL, Cleveland JW, Ikedionwu MO, Carrick JD, Smith JC, Quarles LD (2020). Small molecule FGF23 inhibitors increase serum phosphate and improve skeletal abnormalities in Hyp mice. bioRxiv.

[10] Fischer E, Reuschl S, Quinkler M, Rump LC, Hahner S, Bidlingmaier M, Reincke M, Participants of the German Conn’s Registry—Else Kröner-Fresenius-Hyperaldosteronism Registry (2013). Assay characteristics influence the aldosterone to renin ratio as a screening tool for primary aldosteronism: results of the German Conn’s registry. Horm Metab Res Horm Stoffwechselforschung Horm Metab.

[11] Nikolaeva S, Pradervand S, Centeno G, Zavadova V, Tokonami N, Maillard M, Bonny O, Firsov D (2012). The circadian clock modulates renal sodium handling. J Am Soc Nephrol JASN.

[12] Thosar SS, Rueda JF, Berman AM, Lasarev MR, Herzig MX, Clemons NA, Roberts SA, Bowles NP, Emens JS, Ellison DH, Shea SA (2019). Separate and interacting effects of the endogenous circadian system and behaviors on plasma aldosterone in humans. Am J Physiol-Regul Integr Comp Physiol.

[13] Shackleton C (2010). Clinical steroid mass spectrometry: a 45 year history culminating in HPLC-MS/MS becoming an essential tool for patient diagnosis. J Steroid Biochem Mol Biol.

[14] Kohler H, Hesse RH, Pechet MM (1964). The metabolism of aldosterone. metabolic pathway, isolation, characterization, and synthesis of metabolites. J Biol Chem.

[15] Glatz N, Chappuis A, Conen D, Erne P, Péchère-Bertschi A, Guessous I, Forni V, Gabutti L, Muggli F, Gallino A, Hayoz D, Binet I, Suter P, Paccaud F, Bochud M, Burnier M (2017). Associations of sodium, potassium and protein intake with blood pressure and hypertension in Switzerland. Swiss Med Wkly.

[16] Smith ER, Cai MM, McMahon LP, Holt SG (2012). Biological variability of plasma intact and C-terminal FGF23 measurements. J Clin Endocrinol Metab.

[17] Fuster DG, Morard GA, Schneider L, Mattmann C, Lüthi D, Vogt B, Dhayat NA (2020). Association of urinary sex steroid hormones with urinary calcium, oxalate and citrate excretion in kidney stone formers. Nephrol Dial Transplant Off Publ Eur Dial Transpl Assoc-Eur Ren Assoc.

[18] Dhayat NA, Ackermann D, Pruijm M, Ponte B, Ehret G, Guessous I, Leichtle AB, Paccaud F, Mohaupt M, Fiedler G-M, Devuyst O, Pechère-Bertschi A, Burnier M, Martin P-Y, Bochud M, Vogt B, Fuster DG (2016). Fibroblast growth factor 23 and markers of mineral metabolism in individuals with preserved renal function. Kidney Int.

[19] Levey AS, Stevens LA, Schmid CH, Zhang YL, Castro AF, Feldman HI, Kusek JW, Eggers P, Van Lente F, Greene T, Coresh J, CKD-EPI (Chronic Kidney Disease Epidemiology Collaboration) (2009). A new equation to estimate glomerular filtration rate. Ann Intern Med.

[20] Bingham SA, Cummings JH (1985). The use of creatinine output as a check on the completeness of 24 hour urine collections. Hum Nutr Clin Nutr.

[21] Côté A-M, Firoz T, Mattman A, Lam EM, von Dadelszen P, Magee LA (2008). The 24-hour urine collection: gold standard or historical practice?. Am J Obstet Gynecol.

[22] Forni Ogna V, Ogna A, Vuistiner P, Pruijm M, Ponte B, Ackermann D, Gabutti L, Vakilzadeh N, Mohaupt M, Martin P-Y, Guessous I, Péchère-Bertschi A, Paccaud F, Bochud M, Burnier M, Swiss Survey on Salt Group (2015). New anthropometry-based age- and sex-specific reference values for urinary 24 hour creatinine excretion based on the adult Swiss population. BMC Med.

[23] Dhayat NA, Lüthi D, Schneider L, Mattmann C, Vogt B, Fuster DG (2019). Distinct phenotype of kidney stone formers with renal phosphate leak. Nephrol Dial Transplant Off Publ Eur Dial Transpl Assoc-Eur Ren Assoc.

[24] Dhayat NA, Frey AC, Frey BM, d’Uscio CH, Vogt B, Rousson V, Dick B, Flück CE (2015). Estimation of reference curves for the urinary steroid metabolome in the first year of life in healthy children: tracing the complexity of human postnatal steroidogenesis. J Steroid Biochem Mol Biol.

[25] Ackermann D, Groessl M, Pruijm M, Ponte B, Escher G, d’Uscio CH, Guessous I, Ehret G, Pechère-Bertschi A, Martin P-Y, Burnier M, Dick B, Vogt B, Bochud M, Rousson V, Dhayat NA (2019). Reference intervals for the urinary steroid metabolome: the impact of sex, age, day and night time on human adult steroidogenesis. PLoS ONE.

[26] R: The R Project for Statistical Computing. https://www.r-project.org/. Accessed 4 Dec 2020

[27] Breheny P, Burchett W (2020). Visualization of regression models using visreg. R J.

[28] Perinpam M, Ware EB, Smith JA, Turner ST, Kardia SLR, Lieske JC (2015). Effect of demographics on excretion of key urinary factors related to kidney stone risk. Urology.

[29] Lavi-Moshayoff V, Wasserman G, Meir T, Silver J, Naveh-Many T (2010). PTH increases FGF23 gene expression and mediates the high-FGF23 levels of experimental kidney failure: a bone parathyroid feedback loop. Am J Physiol Renal Physiol.

[30] Masuyama R, Stockmans I, Torrekens S, Van Looveren R, Maes C, Carmeliet P, Bouillon R, Carmeliet G (2006). Vitamin D receptor in chondrocytes promotes osteoclastogenesis and regulates FGF23 production in osteoblasts. J Clin Invest.

[31] Humalda JK, Yeung SMH, Geleijnse JM, Gijsbers L, Riphagen IJ, Hoorn EJ, Rotmans JI, Vogt L, Navis G, Bakker SJL, de Borst MH (2020). Effects of potassium or sodium supplementation on mineral homeostasis: a controlled dietary intervention study. J Clin Endocrinol Metab.

[32] Cogswell ME, Maalouf J, Elliott P, Loria CM, Patel S, Bowman BA (2015). Use of urine biomarkers to assess sodium intake: challenges and opportunities. Annu Rev Nutr.

[33] van Buren M, Rabelink AJ, Bijlsma JA, Koomans HA (1993). Natriuretic and kaliuretic response to potassium load: modulation by sodium intake. Nephrol Dial Transplant Off Publ Eur Dial Transpl Assoc-Eur Ren Assoc.

[34] Simpson SA, Tait JF, Wettstein A, Neher R, Von Euw J, Reichstein T (1953). Isolation from the adrenals of a new crystalline hormone with especially high effectiveness on mineral metabolism. Experientia.

[35] Palmer LG, Frindt G (2000). Aldosterone and potassium secretion by the cortical collecting duct. Kidney Int.

[36] Schück O, Teplan V, Maly J, Franekova J, Malinska H, Stollova M, Latova I, Urbanova J, Skibova J, Viklicky O (2014). The relationship between estimated GFR based on the CKD-EPI formula and renal inulin clearance in potential kidney donors. Clin Nephrol.

[37] Tsuda A, Ishimura E, Uedono H, Yasumoto M, Ichii M, Nakatani S, Mori K, Uchida J, Emoto M, Nakatani T, Inaba M (2016). Comparison of the estimated glomerular filtration rate (eGFR) in diabetic patients, non-diabetic patients and living kidney donors. Kidney Blood Press Res.

[38] Leifheit-Nestler M, Kirchhoff F, Nespor J, Richter B, Soetje B, Klintschar M, Heineke J, Haffner D (2018). Fibroblast growth factor 23 is induced by an activated renin-angiotensin-aldosterone system in cardiac myocytes and promotes the pro-fibrotic crosstalk between cardiac myocytes and fibroblasts. Nephrol Dial Transplant Off Publ Eur Dial Transpl Assoc-Eur Ren Assoc.

[39] Radloff J, Pagitz M, Andrukhova O, Oberbauer R, Burgener IA, Erben RG (2021). Aldosterone is positively associated with circulating FGF23 levels in chronic kidney disease across four species, and may drive FGF23 secretion directly. Front Physiol.

[40] Sharma S, Katz R, Bullen AL, Chaves PHM, de Leeuw PW, Kroon AA, Houben AJHM, Shlipak MG, Ix JH (2020). J Clin Endocrinol Metab.

[41] Abdelhamid S, Blomer R, Hommel G, Haack D, Lewicka S, Fiegel P, Krumme B (2003). Urinary tetrahydroaldosterone as a screening method for primary aldosteronism: a comparative study. Am J Hypertens.

