# Supplementary material for: Urinary tetrahydroaldosterone is associated with circulating FGF23 in kidney stone formers
Source: Urolithiasis. 2022 Feb 24;50(3):333–40. doi: 10.1007/s00240-022-01317-2 (PMC9110437; doi:10.1007/s00240-022-01317-2)
Supplement: Supplementary file 1 — Supplementary file1 (PDF 363 KB) [file 240_2022_1317_MOESM1_ESM.pdf]

Supplementary materials

Urinary tetrahydroaldosterone is associated with circulating FGF23 in kidney stone formers

Matthias B. Moor[1], Nasser A. Dhayat[1], Simeon Schietzel[1], Michael Grössl[1], Bruno Vogt[1], Daniel G. Fuster[1]

[1]Department of Nephrology and Hypertension, Inselspital, Bern University Hospital and Department of Biomedical Research, University of Bern, Bern, Switzerland

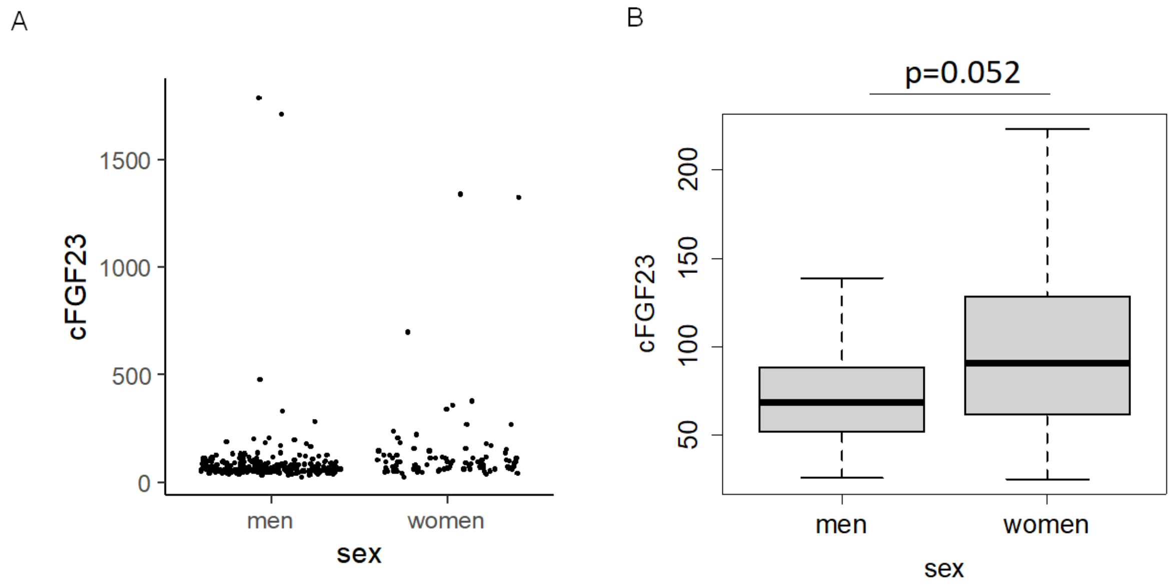

Supplemental Figure 1. A sex-specific analysis of circulating FGF23 in kidney stone formers. Scatterplot (A) and boxplot with whiskers depicting 1.5× interquartile ranges (B) of plasma FGF23 [RU/mL] in men and women.

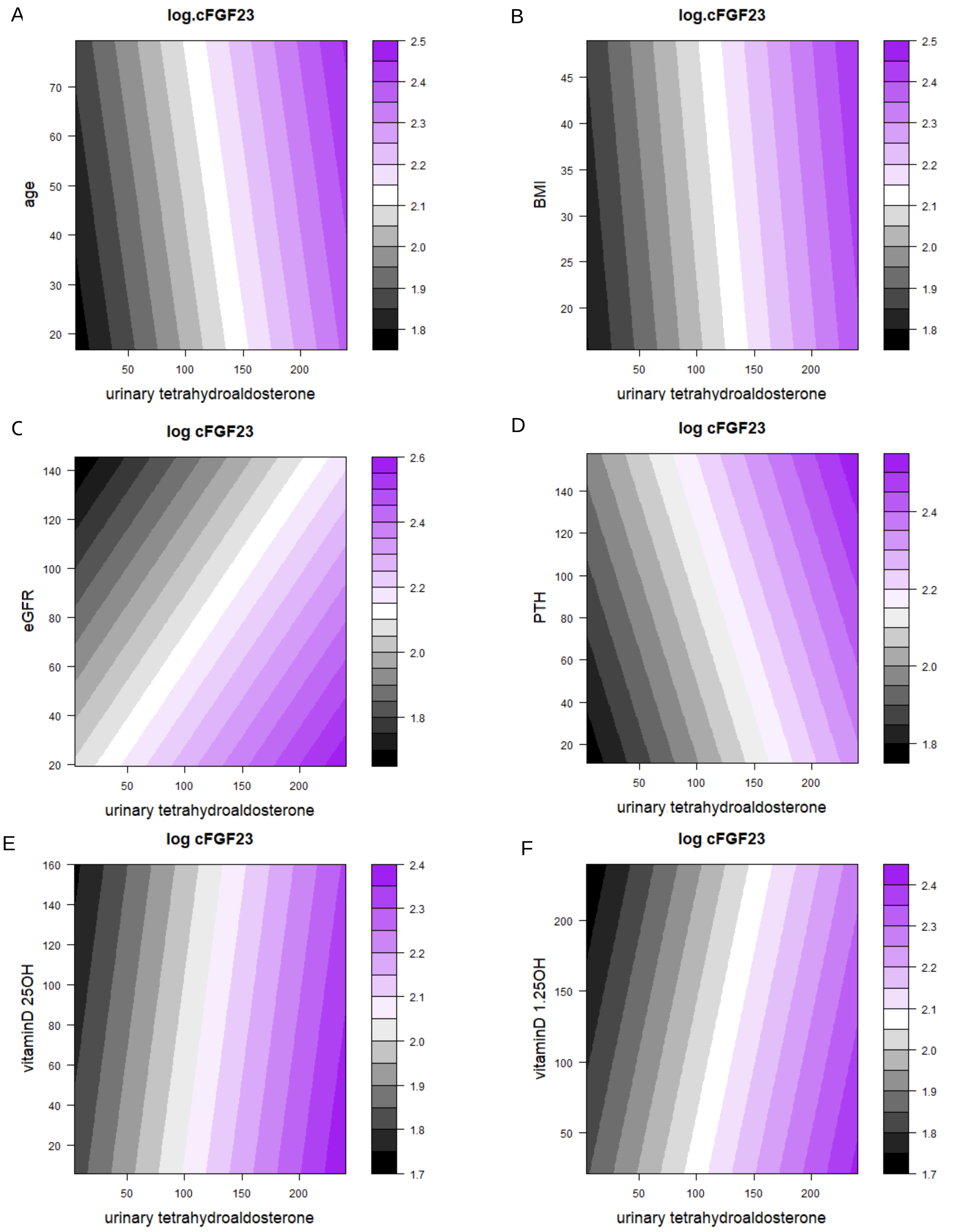

Supplemental Figure 2. Visualization of selected covariables potentially affecting the association between plasma FGF23 and urinary tetrahydroaldosterone. Plots A to F show the multivariable adjusted associations between 24 h urinary tetrahydroaldosterone [µg] and log transformed plasma FGF23 [log RU/mL] and, according to A) age [years], B) BMI [kg/m^2], C) eGFR [mL/min per $1.73m^2$], D) PTH [pg/mL], E) 25(OH) vitamin D [nmol/L], F) 1,25(OH)2 vitamin D [pmol/L]. Except for the respective associated item itself, adjustments were made for A-C) age, sex, BMI and eGFR and D-E) additionally for PTH, 1,25(OH) vitamin D and 1,25OH-vitamin D.

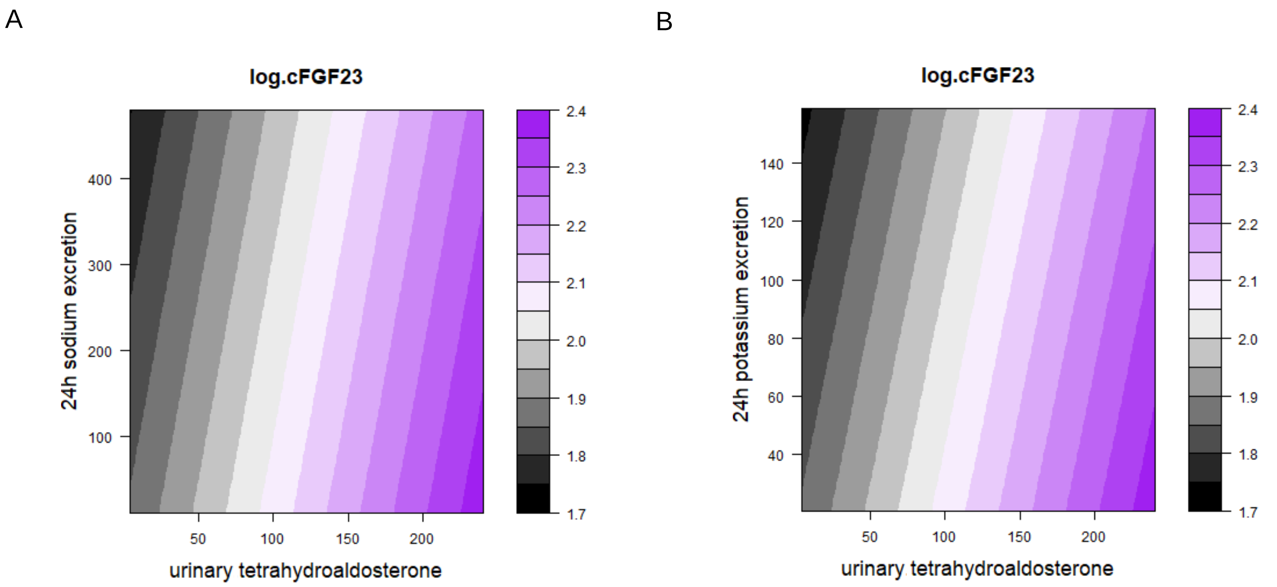

Supplemental Figure 3. Effect of 24 h urinary sodium and potassium excretion on the association between plasma FGF23 and 24 h urinary tetrahydroaldosterone. A and B show the association between 24 h urinary tetrahydroaldosterone excretion [µg] and log transformed circulating plasma concentration of FGF23 [log RU/mL] according to 24 h sodium excretion (A) and 24h potassium excretion (B) in mmol/24 h after adjustment for age, sex, body mass index and eGFR. FGF, fibroblast growth factor. BMI, body mass index. eGFR, estimated glomerular filtration rate.

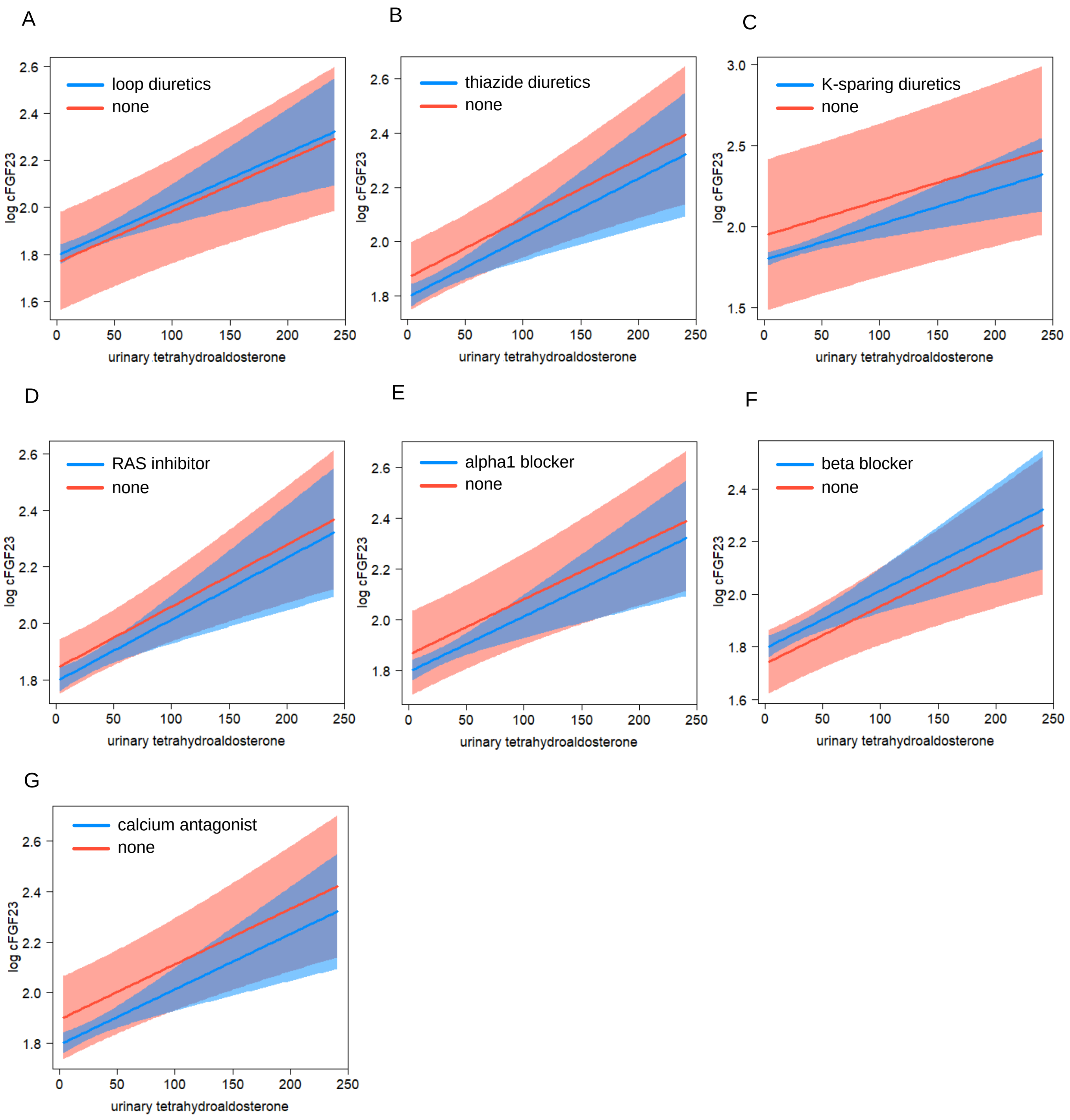

Supplemental Figure 4. Prescribed antihypertensive drugs and the association between urinary tetrahydroaldosterone and log FGF23. Multivariable linear model showing the association between urinary excretion of tetrahydroaldosterone [µg/24 h] and log transformed circulating plasma level of FGF23 [log RU/mL] according to concomitant use of loop diuretics (A), thiazide diuretics (B), K-sparing diuretics (C), RAS inhibitors (D), alpha1 blockers (E), beta blockers (F), and calcium antagonists (G). The model was adjusted for age, sex, body mass index and eGFR. FGF, fibroblast growth factor. BMI, body mass index. RAS, renin angiotensin system. eGFR, estimated glomerular filtration rate CKD-EPI.

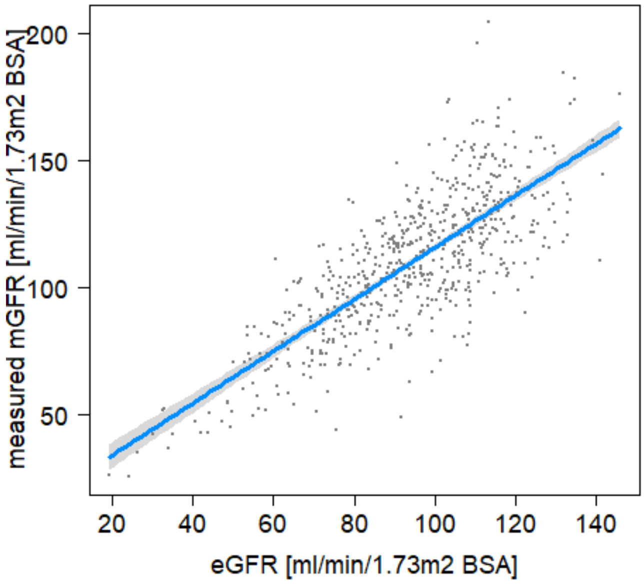

Supplemental Figure 5 Correlation between measured glomerular filtration rate by creatinine clearance (mGFR) and eGFR using the CKD-EPI formula. β: 1.02105, $p=2\times10^{-16}$ and R^2: 0.59. BSA, body surface area.

Supplemental table: Multivariable models models for FGF23 using measured GFR for adjustment

			**multivariable model 1b**		
**Covariable for log cFGF23**	n	β	lower 95%CI	upper 95%CI	p value
urinary tetrahydroaldosterone per log cFGF23 RU/mL increase	314	0.0024884	0.00148446	0.00349238	**0.00000173**

			**multivariable model 2b**		
**Covariable for log cFGF23**	n	β	lower 95%CI	upper 95%CI	p value
urinary tetrahydroaldosterone per log cFGF23 RU/mL increase	295	0.0026026	0.00152326	0.00368191	**0.00000328**

			**multivariable model 3b**		
**Covariable for log cFGF23**	n	β	lower 95%CI	upper 95%CI	p value
urinary tetrahydroaldosterone per log cFGF23 RU/mL increase	314	0.0025184	0.001488	0.0035487	**2.38E-06**

			**multivariable model 4b**		
**Covariable for log cFGF23**	n	β	lower 95%CI	upper 95%CI	p value
urinary tetrahydroaldosterone per log cFGF23 RU/mL increase	313	0.0024652	0.0014328	0.00349764	**4.00E-06**

Model 1b adjusted for age, sex, body mass index, measured glomerular filtration rate. Model 2b adjusted for parameters of model 1b and parathyroid hormone, 25OH-vitamin D and 1.25(OH)2-vitamin D. Model 3b adjusted for age, sex, body mass index, measured glomerular filtration rate, natriuresis, and kaliuresis. Model 4b adjusted for parameters of model 3b and antihypertensives (alpha1 blockers, beta blockers, calcium antagonists, K-sparing diuretics, loop diuretics, thiazides and renin angiotensin system inhibitors).
